# PET/CT vs PET/MRI in Lung Cancer: A Narrative Review of Diagnostic Performance, Practical Trade-offs, and Clinical Impact

**DOI:** 10.7759/cureus.93427

**Published:** 2025-09-28

**Authors:** Basel T Tomalieh, Jessica D Torres Luna, Karthika Murugesan, Areeba Kabir, Delvy Rebellow, Alousious Kasagga, Summayya Anwar

**Affiliations:** 1 Respiratory Medicine, Aintree University Hospital, Liverpool, GBR; 2 Pathology, Universidad Tecnologica Centroamericana (UNITEC), Tegucigalpa, HND; 3 Psychiatry, University of North Texas, Denton, USA; 4 Psychiatry and Behavioral Sciences, California Institute of Behavioral Neurosciences and Psychology, California, USA; 5 Internal Medicine, Malankara Orthodox Syrian Church Medical College, Kolenchery, Kochi, IND; 6 Internal Medicine, California Institute of Behavioral Neurosciences and Psychology, Fairfield, USA; 7 Pathology, Peking University, Beijing, CHN; 8 Biosciences, Commission on Science and Technology for Sustainable Development in the South (COMSATS) University Islamabad, Islamabad, PAK; 9 Research and Development, California Institute of Behavioral Neurosciences and Psychology, Fairfield, USA

**Keywords:** lung cancer staging, non-small cell lung carcinoma (nsclc), pet ct scan, pet mri, small cell lung cancer (sclc), whole-body mri

## Abstract

Lung cancer remains the leading cause of cancer death worldwide. Accurate initial staging is central to treatment selection and prognosis. Hybrid imaging with ^18^F fludeoxyglucose (FDG) positron emission tomography-computed tomography (PET-CT) is widely adopted as the pre-radical staging test, while positron emission tomography-magnetic resonance imaging (PET-MRI) has emerged as an alternative that replaces the CT component with multiparametric MRI, potentially improving soft-tissue assessment and reducing ionising dose. This narrative review evaluates PET-CT and PET-MRI for lung cancer staging across Tumour Node Metastasis (TNM) domains, drawing on English-language studies (2008-2025) with emphasis on head-to-head comparisons in non-small cell lung cancer (NSCLC) and small cell lung cancer (SCLC), complemented by guidelines, quantitative studies and operational literature.

In NSCLC, PET-MRI achieves overall staging performance comparable to PET-CT and improves confidence for pleural, chest-wall and mediastinal invasion, particularly when diffusion-weighted imaging (DWI) and cine/free-breathing radial T1-weighted MRI at 3 T (a higher-field MRI strength than 1.5 T). PET-CT retains an advantage for sub-centimetre and subsolid pulmonary nodules; MRI-led pathways should therefore mandate a contemporaneous thin-slice diagnostic chest CT when such nodules could change intent. Nodal staging is broadly similar between hybrids; where results would alter management, selective invasive confirmation remains appropriate, with combined endobronchial and endoscopic ultrasound (EBUS-EUS) improving the sampling efficiency. For distant disease, PET-CT is an efficient whole-body screen at baseline, while PET-MRI adds value for brain and liver problem-solving. In SCLC, a prospective cohort reported higher T-category and overall TNM accuracy with whole-body MRI and coregistered PET-MRI than with PET-CT.

Quantitatively, standardised uptake values (SUVs) correlate between platforms but are not interchangeable; centres should harmonise using European Association of Nuclear Medicine Research Ltd (EARL) and Quantitative Imaging Biomarkers Alliance (QIBA) guidance and document MRI attenuation-correction choices (e.g., Dixon vs ultrashort/zero echo time (UTE/ZTE)). Operationally, PET-MRI reduces anatomic radiation but typically requires longer slots and specialised workflow; PET-CT is faster, widely available, and linked to fewer futile thoracotomies and favourable cost-effectiveness.

Overall, PET-CT should remain the default hybrid, with PET-MRI deployed selectively, particularly for complex local invasion, integrated brain-to-body staging, or dose reduction, while safeguarding small-nodule detection. Priorities include multicentre trials with harmonised protocols, management-impact endpoints, and explicit rules for integrating chest CT into MRI-led pathways.

## Introduction and background

**Figure 1:**
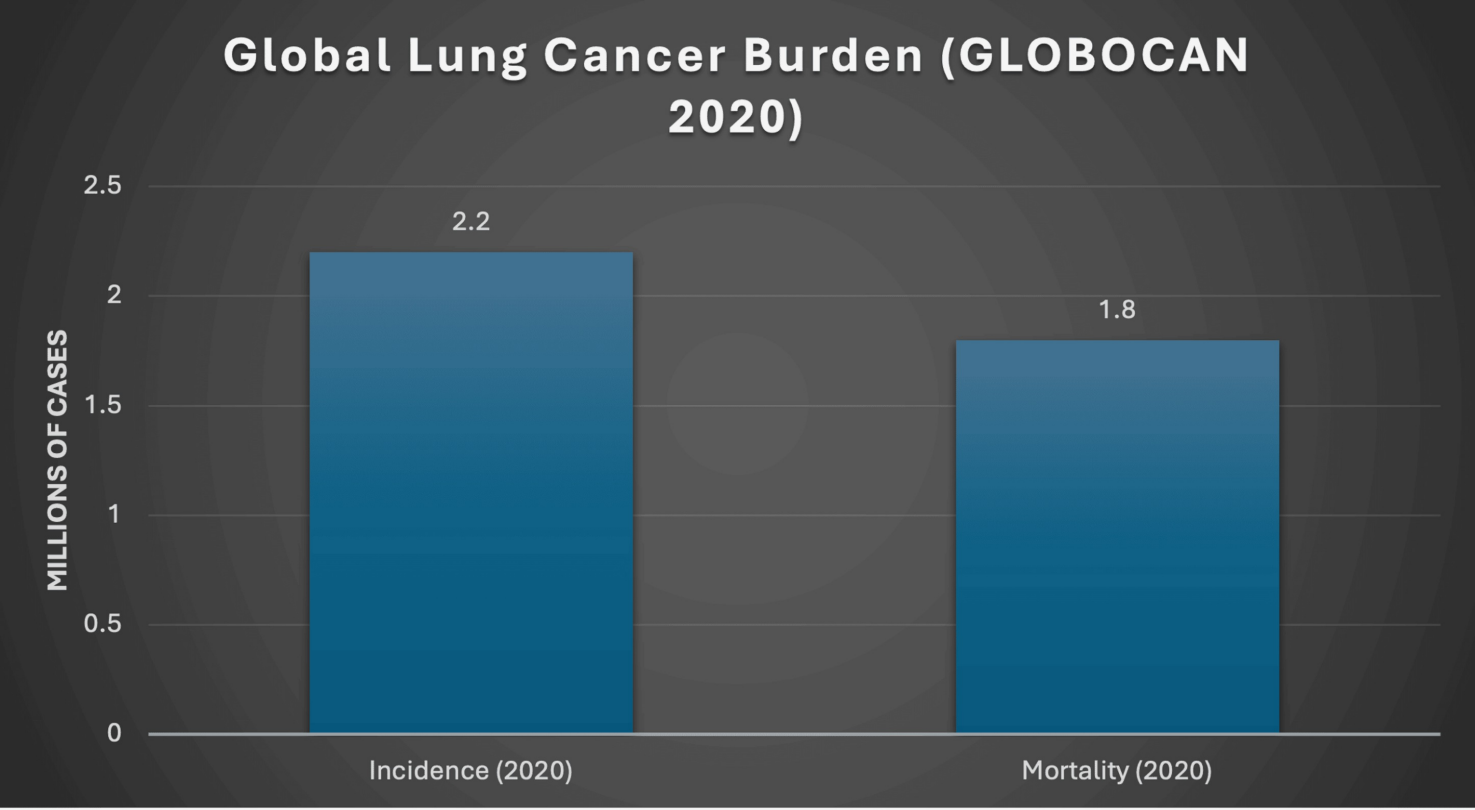
Global lung cancer incidence and mortality in 2020 (millions): GLOBOCAN estimate Source: Ref. [1].

Lung cancer is the leading cause of cancer death worldwide. In 2020, there were approximately 2.2 million new lung cancer cases (Figure 1). Many patients still present with advanced disease at diagnosis, making accurate initial staging essential [1]. Staging is organised by the Tumour Node Metastasis (TNM) system. The International Association for the Study of Lung Cancer is updating the evidence base for the ninth edition so that stage groups reflect current outcomes and larger datasets [2]. UK guidance places imaging at the centre of care: The National Institute for Health and Care Excellence (NICE) sets out how and when to stage, and the Royal College of Radiologists (RCR) provides practical recommendations for consistent imaging pathways [3,4]. A concise summary of the same pathway appears in the *British Medical Journal* (BMJ) update, reinforcing PET-CT as the pre-radical staging checkpoint in UK practice [5].

For the last two decades, ^18^F fludeoxyglucose (FDG) PET-CT has been the standard hybrid test before radical treatment. Randomised and prospective trials show that adding PET-CT reduces futile surgery and improves the selection for radical therapy compared with conventional staging alone, and economic analyses show that this approach is cost-effective [6,7]. These data support guideline advice to confirm stage with PET-CT before surgery or chemoradiotherapy and to use results to guide multidisciplinary planning, including invasive nodal sampling when appropriate [3,4].

PET-CT still has limits. The CT part adds ionising radiation; soft-tissue contrast can be modest for questions such as chest-wall or mediastinal invasion and for liver or marrow assessment; and small brain metastases usually need a dedicated brain MRI [4,8]. These gaps have led to growing use of PET-MRI, which combines whole-body PET with multiparametric MRI (including diffusion-weighted imaging) and removes the extra CT dose. An international consensus now gives practical guidance for PET-MRI protocols and highlights issues such as MRI-based attenuation correction and workflow that centres need to plan for [8,9]. Editorial commentary has also argued that whole-body MRI can strengthen lung cancer staging when soft-tissue detail is decisive [10].

Head-to-head studies in non-small cell lung cancer (NSCLC) report that PET-MRI achieves overall staging performance similar to PET-CT, and MRI-based protocols can improve assessment of local soft-tissue invasion at the pleura, chest wall and mediastinum [11-15]. At the same time, MRI is less sensitive to very small intrapulmonary nodules: direct comparisons have shown sub-centimetre nodules seen on CT but not on PET-MRI or whole-body MRI, which can occasionally change T or M category [16,17]. Evidence in small cell lung cancer (SCLC), although more limited, points in the same direction for local staging. In a prospective cohort, whole-body MRI and coregistered PET-MRI outperformed PET-CT for T category and for overall TNM accuracy, while all advanced modalities did better than conventional staging for most endpoints [18].

This review compares PET-CT and PET-MRI in lung cancer. We focus on diagnostic performance across T, N and M categories, practical trade-offs (radiation, protocol design, scan duration and availability), and what these findings mean for multidisciplinary decisions. We place results in the context of UK guidance and international consensus, and we rely on prospective head-to-head studies in NSCLC and SCLC to show where PET-MRI can complement or, in selected settings, substitute for PET-CT without loss of diagnostic quality [3,9,11,18].

## Review

Summary of evidence and scope

PET-CT and PET-MRI are central imaging options for lung cancer staging. PET-CT remains the guideline standard, while PET-MRI offers higher soft-tissue contrast with lower anatomic radiation dose, supported by consensus recommendations [3,4,8,9]. Across head-to-head NSCLC studies, PET-MRI achieves overall staging performance comparable to PET-CT and can improve the assessment of pleural, chest-wall, and mediastinal invasion [11-15]. At the same time, CT retains better sensitivity for sub-centimetre pulmonary nodules [16,17]. In SCLC, a prospective cohort reported higher accuracy with whole-body MRI and coregistered PET-MRI than with PET-CT for T category and overall TNM stage, while all advanced modalities outperformed conventional staging on several endpoints [18]. Given this evidence and service considerations, the following sections examine diagnostic performance across T, N, and M categories, operational trade-offs (radiation, protocols, scan duration, access), and effects on multidisciplinary decisions, while briefly appraising study quality and identifying research gaps.

T-staging: local tumour extent and soft-tissue planes

Prospective NSCLC comparisons show that PET-MRI matches PET-CT overall and can improve confidence when assessing pleural, chest-wall, or mediastinal invasion. Studies by Huellner et al., Lee et al., Heusch et al., and Kirchner et al. reported broadly similar patient-level TNM accuracy, with MRI-containing protocols often helping at T3-T4 thresholds where tissue planes matter [11-14]. A three-way *American Journal of Roentgenology* (AJR) study that compared whole-body MRI, coregistered PET-MRI (1.5 T and 3 T), and integrated PET-CT reported at least comparable overall accuracy for MRI-containing pathways and better depiction of complex local invasion in selected cases [15]. Here, 1.5 T and 3 T are MRI field strengths; 3 T provides higher signal-to-noise and finer resolution for pleural, chest-wall and mediastinal assessment but can be more artefact-prone. Related thoracic work in malignant pleural mesothelioma showed that MRI-containing pathways better capture pleural and chest-wall extent than PET-CT, supporting MRI’s advantage at soft-tissue interfaces [19].

The SCLC cohort showed the same direction of effect for local staging. Whole-body MRI and coregistered PET-MRI achieved 94.9% accuracy for T, higher than PET-CT (85.7%), and this carried through to overall TNM accuracy (88.8% and 86.7% vs 77.6%) [18]. This likely reflects MRI’s ability to show soft-tissue boundaries and airway or mediastinal involvement that can be equivocal on low-dose CT used within PET-CT protocols [18]. UK guidance recognises the complementary roles: PET-CT remains the backbone, with MRI used when it will change planning for surgery or radiotherapy [3,4].

Beyond aggregate accuracy, protocol-dependent MRI techniques sharpen T-category assessment at key interfaces. Contemporary thoracic PET-MRI protocols using diffusion-weighted imaging, dynamic contrast and cine or free-breathing radial T1, often at 3 T, can reduce equivocal calls for visceral pleural and chest-wall invasion, with the greatest gains when T3-T4 thresholds hinge on tissue planes [20,21]. These gains are not inherent to MRI as a class: performance varies with field strength, motion management and attenuation-correction workflow, which likely contributes to heterogeneity across studies [20,22]. In practice, when the pre-test probability of chest-wall or mediastinal invasion is high, MRI-containing pathways can support resectability decisions, while CT remains useful for cortical bone detail [22].

N-staging: mediastinal and hilar nodes

Because FDG uptake drives a large part of nodal assessment in both hybrids, prospective NSCLC studies usually show similar N-staging performance for PET-MRI and PET-CT when appropriate reference standards are used [11-14], and a recent meta-analysis confirms no material difference between PET-MRI and PET-CT for nodal metastasis detection in NSCLC [23]. Independent PET-CT series also document strong nodal staging performance and better agreement with pathology than CT alone, including preoperative comparisons demonstrating higher accuracy of PET/CT versus contrast-enhanced CT [24-26]. Where nodal results will alter management, invasive confirmation remains important; this principle is supported by prospective and randomised work and is reflected in practice guidance [3,27,28]. Pooled data also show that combined endobronchial and endoscopic ultrasound (EBUS+EUS) improves mediastinal nodal staging performance compared with either alone, supporting selective invasive confirmation when imaging results will alter intent [29]. Broader overviews of PET-CT in NSCLC reach similar conclusions for nodal assessment and pathway positioning [30]. In SCLC, PET-CT is valuable for baseline staging but mediastinal nodal accuracy is imperfect, and selective invasive sampling remains advisable when results would change chemoradiotherapy fields [31].

Taken together, nodal performance is modality-balanced between PET-MRI and PET-CT when robust reference standards are used, but management-level specificity is best preserved by a strategy that channels PET-positive or equivocal stations to targeted EBUS+EUS rather than routine systematic sampling [23,29]. In SCLC, the same principle applies: PET-CT improves staging and planning, yet selective invasive confirmation remains important when results will redirect radiotherapy volumes or alter systemic therapy [31].

M-staging: brain, liver, bone and adrenal

MRI performs well in the brain, liver and bone marrow, where soft-tissue contrast and diffusion-weighted imaging aid detection and characterisation. For the brain, dedicated MRI remains the reference standard for detecting micrometastases and for treatment planning, outperforming CT-based components in hybrid protocols [32]. For the liver, multiparametric MRI (including diffusion-weighted and, where appropriate, hepatobiliary contrast sequences) increases sensitivity for sub-centimetre deposits and reduces indeterminate findings compared with CT and FDG PET, supporting its use for problem-solving within PET-MRI pathways [33]. Whole-body and three-way thoracic comparisons report parity or superiority of MRI-containing pathways for metastatic assessment at these sites, with PET adding metabolic context to resolve indeterminate lesions [11,12,15,34].

A meta-analysis across malignancies likewise suggests comparable or improved detection of distant disease with PET-MRI relative to PET-CT in appropriate contexts [35]. Conversely, at initial staging in NSCLC, PET-CT shows high whole-body performance for distant metastases (pooled area under the curve around 0.97 with specificity ≈0.96), reinforcing its role as the default screen when rapid triage is required [36]. In SCLC, whole-body MRI and PET-MRI matched PET-CT for M-category accuracy and were better than conventional staging [18]. In practice, combining metabolic information from PET with targeted MRI problem-solving remains a pragmatic approach when marrow-only disease is suspected. Taken together, these data support a pragmatic division of labour: PET-CT for efficient whole-body exclusion of distant disease, with MRI leveraged for high-yield sites, particularly brain and liver, when lesion conspicuity or characterisation will influence treatment intent [32,33,36].

Outside lung cancer, multiple cohorts report that PET-MRI can improve soft-tissue lesion conspicuity and inform management in the head and neck, gynaecologic, colorectal, breast, and for intracranial masses [37-41]. However, site-specific differences remain: in differentiated thyroid cancer, PET-CT has outperformed PET-MRI, underscoring that superiority is not universal [42]. Skeletal assessment also varies by disease; a network analysis in prostate cancer highlights how modality and tracer choice can shift apparent performance for bone metastases, a caution when extrapolating across tumours [43].

Detecting small pulmonary nodules

A consistent limitation of thoracic MRI is reduced visibility for sub-centimetre nodules. Paired studies show nodules visible on CT or PET-CT but not on MRI or PET-MRI; a minority later prove malignant and can affect staging. Sawicki and colleagues reported outcomes of nodules missed on PET-MRI that were seen on PET-CT, quantifying the small but relevant risk of upstaging from very small lesions [16]. A companion analysis compared PET-MRI with PET-CT for pulmonary lesion detection and characterisation, again confirming CT’s advantage for small parenchymal nodules, particularly for sub-centimetre and ground-glass/part-solid nodules [44]. Trimodality work comparing low-dose CT with Dixon-based MRI showed higher detection rates and fuller size coverage with CT [17]. Rauscher and colleagues reached similar conclusions, reinforcing the need to protect small-nodule sensitivity when MRI-led pathways are used [45]. A practical safeguard is to ensure a recent diagnostic-quality chest CT (or an adjunct low-dose CT) whenever sub-centimetre intrapulmonary disease could change treatment intent [3,4,16,17,44,45]. This limitation is most pronounced for subsolid and <10 mm nodules, so MRI-led pathways intended for curative candidates should mandate contemporaneous thin-slice diagnostic chest CT.

Quantitative metrics and technical factors

Standardised uptake value (SUV) measurements on PET-MRI and PET-CT correlate well, but they are not interchangeable. Differences in MRI-based attenuation correction (AC), scatter modelling, timing and time-of-flight (TOF)/point-spread function (PSF) availability can cause small but systematic offsets, so PET-CT cut-offs should not be applied directly to PET-MRI without local validation [11,14,46,47]. Harmonisation frameworks such as the European Association of Nuclear Medicine Research Ltd (EANM EARL) and QIBA FDG PET-CT profile provide practical standards to improve cross-centre comparability and define precision claims for clinical and trial use [48,49]. Tracer and protocol differences can further limit direct translation of thresholds; early PET-MRI experience with fibroblast-activation protein tracers shows a behaviour distinct from FDG, reinforcing the need for centre-specific validation [50]. For PET-MRI specifically, advances in MRI-based AC, including ultrashort echo time (UTE) and zero echo time (ZTE) bone modelling, atlas-based and machine-learning (ML) approaches, can reduce bias but remain heterogeneous across vendors and sites [51]. Implementation choices also matter. Field strength (1.5 T vs 3 T), the quality of diffusion-weighted imaging (DWI), motion control, contrast timing and the interval between paired scans can each tilt results. The AJR three-way comparison explicitly tested both 1.5 T and 3 T systems, and several NSCLC studies note that protocol discipline and reader experience are key to achieving parity between hybrids [12-15]. In SCLC, PET and MRI were coregistered using a non-rigid algorithm; the process was fast and accurate in that study, but results could differ with other software or with integrated PET-MRI scanners [18].

In practice, ensuring transportable SUVs requires harmonised acquisition and reconstruction (EARL) and adherence to quantitative profiles (QIBA) so that thresholds and longitudinal changes are interpretable across platforms and time [48,49]. For MRI-led pathways, AC method (e.g., Dixon versus ultrashort/zero echo time (UTE/ZTE) and ML), field strength, and TOF/PSF availability should be documented and locally validated before applying PET-CT-derived cut-offs, as these factors remain major sources of between-study variability [51].

Radiation, workflow and patient experience

Replacing CT with MRI removes the anatomic CT dose, which is helpful for younger patients and those needing repeated restaging. Consensus and review articles highlight this advantage but also point to longer acquisition times and more complex workflows that can affect throughput and access [8,9]. Real-world mixed oncology series shows that PET-MRI can inform management in a way comparable to PET-CT while also describing operational constraints (slot length, staff expertise) that services should plan for [47,52]. In the SCLC cohort, mean interpretation time was shortest for whole-body MRI compared with PET-MRI, PET-CT and conventional staging, although this reflects a specific protocol and may not generalise to all centers [18].

Impact on clinical management

The strong role of PET-CT in lung cancer rests on outcomes as well as accuracy. A landmark randomised trial showed that adding PET-CT to conventional preoperative staging reduced futile thoracotomies, establishing PET-CT as standard practice for surgical candidates [7]. A linked economic analysis showed that PET-CT-guided pathways are cost-effective [6]. In SCLC specifically, an analysis from the CONVERT randomised trial underscored the value of PET-CT for accurate staging and planning within chemoradiotherapy pathways [53]. In thoracic oncology, one prospective study found that substituting PET-MRI for PET-CT rarely changed treatment plans, suggesting practical equivalence when protocols and expertise are robust [54]. In a prospective mixed-oncology cohort (330 same-day examinations), PET-MRI yielded additional management-relevant findings in 8% of patients, predominantly brain and liver, albeit with higher per-examination costs than PET-CT [52]. Guidance reflects these lessons by recommending PET-CT before radical therapy and advocating invasive nodal assessment when imaging results will alter intent [3,4,27,28].

Patient subgroups and practical use

A tailored approach is sensible. PET-CT should remain the default hybrid in most NSCLC pathways because it is widely available, fast, and backed by management and economic evidence [3,4,6,7]. PET-MRI is a reasonable alternative in centres with optimised thoracic protocols when: (i) the key question is pleural, chest-wall or mediastinal invasion, (ii) brain-to-pelvis imaging in a single visit is desirable or (iii) reducing radiation is important in younger patients or during long-term surveillance. Operationally, in MRI-led pathways mandate a contemporaneous thin-slice diagnostic chest CT when sub-centimetre disease could change intent, prefer PET-CT when MRI is contraindicated or rapid throughput is required, and in SCLC retain selective invasive nodal confirmation when results would alter chemoradiotherapy fields [3,4,8,13-18,44,45].

**Table 1:** Hybrid PET Imaging and Clinical Implications for Lung Cancer Staging - Study Designs, Key Findings, and Practice Takeaways PET: Positron emission tomography; CT: computed tomography; MRI: magnetic resonance imaging; PET/CT: PET with CT; PET/MRI: PET with MRI; WB-MRI: whole-body MRI; NSCLC: non-small cell lung cancer; SCLC: small cell lung cancer; TNM: tumour-node-metastasis; DWI: diffusion-weighted imaging; SUV: standardised uptake value; AC: attenuation correction; MDT: multidisciplinary team; EBUS/EUS: endobronchial/endoscopic ultrasound.

Clinical Question	Study (Reference)	Design & Comparison	Main Finding	Practical Takeaway
Overall NSCLC staging: PET/MRI vs PET/CT	Huellner et al. 2016 [11]	Prospective head-to-head NSCLC; PET/MRI vs PET/CT	Comparable overall TNM staging performance	PET/MRI can substitute for PET/CT where protocols and expertise are robust
	Lee et al. 2016 [12]	Prospective pre-op NSCLC; PET/MRI vs PET/CT	Similar staging performance; MRI helpful for local invasion in selected cases	Consider PET/MRI when local invasion detail is pivotal pre-op
	Heusch et al. 2014 [13]	Prospective NSCLC; PET/MRI vs PET/CT	Similar thoracic staging performance	Either hybrid acceptable for thoracic staging
	Kirchner et al. 2019 [14]	Prospective NSCLC; PET/MRI vs PET/CT	Comparable T/N/M accuracy	Supports practical interchangeability
Local invasion (T3–T4 interfaces)	Ohno et al. 2020 AJR (three-way) [15]	WB-MRI & coreg PET/MRI (1.5/3T) vs PET/CT	MRI-containing pathways ≥ comparable overall; better depiction of complex soft-tissue invasion in selected cases	Use MRI-containing protocols when pleura/chest-wall/mediastinal planes determine resectability
	Ohno et al. 2019 AJR (MPM) [19]	Mesothelioma; WB-MRI/PET-MRI vs PET/CT	MRI better captures pleural/chest-wall extent	Cross-thoracic signal reinforces MRI’s soft-tissue edge
	Ohno et al. 2023 [20]	State-of-the-art MR review for lung cancer TNM	Protocol-dependent gains for pleural/chest-wall/mediastinal invasion (DWI, cine, 3 T)	Optimise MRI sequences/field strength when T3–T4 thresholds hinge on tissue planes
	Zhang et al. 2021 (KJR) [21]	CT vs contrast-enhanced radial T1 3-T MRI for VPI assessment.	Improved characterisation of visceral pleural/chest-wall invasion with radial/cine MRI	Use targeted MRI when VPI or chest-wall abutment is equivocal
SCLC initial staging	Ohno et al. 2022 AJR [18]	Prospective; conventional tests vs PET/CT vs WB-MRI vs coreg PET/MRI	WB-MRI & PET/MRI higher T and overall TNM accuracy than PET/CT; all advanced tests > conventional	Integrate MRI at baseline SCLC staging to optimise T and TNM
Nodal staging	Ceylan et al. 2012 [26]	Contrast-enhanced CT vs PET/CT (pre-op NSCLC)	PET/CT superior to contrast-enhanced CT for N staging	PET/CT remains backbone for mediastinum; confirm invasively if management changes
	Darling et al. 2011 (ELPET) [27]	PET/CT vs invasive mediastinal staging	PET/CT improves over CT alone but does not obviate invasive confirmation	Combine PET/CT with EBUS/EUS when results alter intent
	Zhang et al. 2024 [23]	NSCLC; PET/MRI vs PET/CT for nodal metastasis	PET/MRI ≈ PET/CT for N staging on pooled analysis	Supports modality balance; choose based on logistics/expertise
	Liu et al. 2022 (Front Med) [29]	Systematic review/meta-analysis of combined EBUS+EUS	Combined endosonography > either alone for mediastinal staging	Target PET-positive/equivocal stations for selective invasive confirmation
	Hockmann et al. 2023 (SCLC) [31]	Limited-disease SCLC; PET/CT nodal staging accuracy	Useful but imperfect mediastinal N staging; sampling still needed when results change fields	Retain selective invasive nodal confirmation in SCLC pathways
Distant metastases (brain/liver/bone)	Sommer et al. 2012 [34]	WB-DWI-MRI vs PET/CT (pre-op NSCLC)	Comparable pre-op staging; potential advantages at soft-tissue sites	MRI-containing protocols helpful where liver/marrow detail matters
	Zhang et al. 2023 [35]	Mixed-oncology PET/MRI vs PET/CT	Comparable or improved distant-met detection context-dependently	PET/MRI acceptable for M-staging in selected settings
	Derks et al. 2022 (Br J Radiol) [32]	Review of brain metastasis imaging	MRI is reference standard for micrometastases and planning	Use dedicated brain MRI/MRI-led pathways when brain disease is plausible
	Maino et al. 2023 (World J Radiol) [33]	Review of liver metastasis imaging	MRI (DWI ± hepatobiliary contrast) improves sub-cm detection and reduces indeterminates	Leverage MRI for liver problem-solving within hybrid pathways
	Yu et al. 2018 (Cancer Manag Res) [36]	NSCLC meta-analysis of PET/CT for distant metastases	High whole-body performance (AUC ≈ 0.97; high specificity)	Keep PET/CT as default rapid whole-body screen at baseline
Small pulmonary nodules	Sawicki et al. 2016 (outcomes) [16]	Nodules missed on PET/MRI vs seen on PET/CT	A minority of missed sub-cm nodules were malignant → occasional upstaging	If sub-cm disease could change intent, add/review diagnostic chest CT with PET/MRI
	Sawicki et al. 2016 (detection) [44]	PET/MRI vs PET/CT	CT better for small parenchymal nodules	Protect small-nodule sensitivity with CT
	Stolzmann et al. 2013 [17]	Trimodality PET/CT-MR: low-dose CT vs Dixon-MR	Low-dose CT detects more and smaller nodules than MR	Do not rely on MR alone for micro-nodules
	Rauscher et al. 2014 [45]	PET/MR vs PET/CT (pulmonary lesions)	Confirms CT’s edge for tiny nodules; technical notes	Pair PET/MRI with recent chest CT when relevant
Quantitation (SUVs)	Al-Nabhani et al. 2014 [46]	Clinical PET/CT vs PET/MR (qual/quant)	SUVs correlate but are not interchangeable	Avoid copying PET/CT cut-offs to PET/MR without local validation
	Drzezga et al. 2012 [47]	Early integrated PET/MR vs PET/CT	Broad comparability; early quantitation caveats	Anticipate small systematic SUV offsets
	Aide et al. 2017 (EANM EARL) [48]	Quantification harmonisation framework	Standardises acquisition/reconstruction to improve comparability	Adopt EARL protocols when thresholds/longitudinal changes guide care
	Kinahan et al. 2020 QIBA FDG Profile [49]	Quantitative imaging profile for FDG PET/CT	Defines precision claims and standard operating steps	Use QIBA-aligned workflows in trials and quantitative clinical pathways
	Krokos et al. 2023 Review (EJNMMI Phys) [51]	MR-based AC methods (Dixon, UTE/ZTE, atlas/ML)	AC choice can shift SUVs; vendor/site heterogeneity persists	Document and locally validate AC approach before applying PET/CT-derived thresholds
Tracers / sequences (caution)	Qin et al. 2022 (FAPI vs FDG) [50]	FAPI-PET/MR vs FDG PET/CT (gastric)	Different tracer behaviour	Validate thresholds per tracer & platform
Workflow, dose, management impact	Mayerhoefer et al. 2020 [52]	Prospective 330 exams; PET/MRI vs PET/CT	Management impact comparable; operational/cost considerations	PET/MRI feasible; plan for slot time/workflow
	Schaarschmidt et al. 2017 [54]	NSCLC; PET/MR vs PET/CT	Therapy plans rarely changed by swapping modalities	Practical equivalence in real-world MDTs
Health-economic and outcome anchors	Fischer et al. 2009 NEJM [7]	RCT: add PET/CT to conventional pre-op staging	Fewer futile thoracotomies	PET/CT as standard pre-radical checkpoint
	Søgaard et al. 2011 [6]	Cost-effectiveness alongside RCT	PET/CT staging pathway cost-effective	Economic rationale for default PET/CT
	Manoharan et al. 2019 (CONVERT) [53]	SCLC chemoradiation RCT analysis	PET/CT supports accurate staging/planning	Reinforces PET/CT role in SCLC pathways

Brief appraisal of study quality

Overall, the lung-specific head-to-head evidence is moderate in quality. Strengths include prospective, within-patient designs, explicit T/N/M endpoints, and the use of histology or composite clinical standards in several cohorts [11-15,18,19]. The main limitations are the predominance of single-centre studies with modest sample sizes and protocol heterogeneity (field strength, DWI quality, contrast timing, motion control, scan interval), which likely drives between-study variability [11-15,18-20]. For PET-MRI in particular, variability in MRI-based attenuation correction (Dixon vs UTE/ZTE or learning-based methods) and differences in TOF/PSF and reconstruction can shift SUVs, limiting the transportability of thresholds across platforms and centres [46-49,51]. The risks of verification bias and spectrum effects persist because invasive nodal sampling was not uniform and reference standards differed by site; some paired scans were non-contemporaneous, introducing biological and technical noise. Mixed-oncology series inform operational outcomes but reduce lung-specific generalisability [47,52,55]. Meta-analyses support modality balance for nodal staging (PET-MRI ≈ PET-CT) and comparable distant-disease detection in appropriate contexts, but heterogeneity remains substantial [23,27]. Finally, the randomised and economic trials that established PET-CT’s management benefits pre-date contemporary PET-MRI and cannot by themselves determine one-for-one substitution [6,7]. Taken together, these factors justify our emphasis on context- and protocol-dependent performance, local validation when applying PET-CT-derived cut-offs to PET-MRI, and harmonised quantitation when thresholds or longitudinal changes inform decisions [48,49].

Limitations of this review (search and selection)

We performed a narrative search of PubMed for human, English-language studies published from 2008 to 2025. PubMed filters applied: Clinical Study, Comparative Study, Journal Article; Species: Humans; Language: English; Publication dates: 2008-2025. Core inclusion criteria were prospective or retrospective clinical studies evaluating PET-CT, PET-MRI, or whole-body MRI for lung cancer (NSCLC or SCLC) staging, with preference for within-patient head-to-head designs. In addition, where directly relevant to staging performance, quantitation, or workflow, we considered recent systematic reviews/meta-analyses and technical or consensus reviews to contextualise protocol choices and management impact. We excluded case reports, paediatric or animal studies, editorials, and non-comparative technical notes. Representative queries included: (“PET/MRI” OR “PET-MR”) AND (lung OR NSCLC) AND (staging OR TNM); (“PET/CT” OR “PET-CT”) AND (lung OR NSCLC) AND (staging OR TNM); and (“PET/MRI” OR “PET-MR”) AND (“PET/CT” OR “PET-CT”) AND (cancer OR tumour) to capture cross-oncology operational data. We also used targeted terms for nodal staging (including SCLC), small pulmonary nodules, brain and liver metastases, management impact and cost-effectiveness, and quantitative/technical topics such as attenuation correction and harmonisation. Last search: September 4, 2025.

## Conclusions

In conclusion, this review clarifies how PET-CT and PET-MRI fit into lung cancer staging. PET-CT remains the default because it is fast, widely available, and linked to better surgical selection and cost-effectiveness. PET-MRI achieves similar overall staging performance while offering higher soft-tissue contrast and integrated brain-to-body assessment; its value is greatest for suspected pleural, chest-wall, or mediastinal invasion and for brain or liver problem-solving. A consistent limitation is reduced sensitivity for very small or subsolid pulmonary nodules, so MRI-led pathways should be paired with a contemporaneous diagnostic-quality chest CT whenever sub-centimetre disease could change intent.

Practically, use PET-CT as the standard pre-radical staging test and deploy PET-MRI selectively where its advantages matter most or dose reduction is a priority, while safeguarding small-nodule detection. Nodal staging performance is broadly comparable between hybrids; invasive confirmation remains important when results will alter management, with combined EBUS/EUS a sensible targeted strategy. In SCLC, whole-body MRI and PET-MRI can improve T category and overall TNM accuracy at baseline, but selective invasive nodal confirmation is still appropriate when findings would change chemoradiotherapy fields. Quantitatively, centres should avoid transplanting PET-CT thresholds to PET-MRI without local validation, adopt harmonised protocols, and document attenuation-correction choices to ensure interpretable SUVs. Priorities now are multicentre trials with harmonised protocols and management-impact endpoints, clearer rules for integrating chest CT into MRI-led pathways, and realistic modelling of throughput and access.
